# Molecular and cellular correlates of the CIITA-mediated inhibition of HTLV-2 Tax-2 transactivator function resulting in loss of viral replication

**DOI:** 10.1186/1479-5876-9-106

**Published:** 2011-07-07

**Authors:** Chiara Orlandi, Greta Forlani, Giovanna Tosi, Roberto S Accolla

**Affiliations:** 1 Department of Experimental Medicine, University of Insubria, Varese, Italy

## Abstract

**Background:**

MHC class II transactivator CIITA inhibits the function of HTLV-2 Tax-2 viral transactivator and, consequently, the replication of the virus in infected cells. Moreover overexpression of the nuclear factor NF-YB, that cooperates with CIITA for the expression of MHC class II genes, results also in inhibition of Tax-2 transactivation. The purpose of this investigation was to assess the cellular and molecular basis of the CIITA-mediated inhibition on Tax-2, and the relative role of NF-YB in this phenomenon.

**Methods:**

By co-immunoprecipitation of lysates from 293T cells cotransfected with CIITA or fragments of it, and Tax-2 it was assessed whether the two factors interact *in vivo*. A similar approach was used to assess Tax-2-NF-YB interaction. In parallel, deletion fragments of CIITA were tested for the inhibition of Tax-2-dependent HTLV-2 LTR-luciferase transactivation. Subcellular localization of CIITA and Tax-2 was investigated by immunofluorescence and confocal microscopy.

**Results:**

CIITA and Tax-2 interact *in vivo *through at least two independent regions, at the 1-252 N-term and at the 410-1130 C-term, respectively. Interestingly only the 1-252 N-term region mediates Tax-2 functional inhibition. CIITA and Tax-2 are localized both in the cytoplasm and in the nucleus, when separately expressed. Instead, when coexpressed, most of Tax-2 colocalize with CIITA in cytoplasm and around the nuclear membrane. The Tax-2 minor remaining nuclear portion also co-localizes with CIITA. Interestingly, when CIITA nucleus-cytoplasm shuttling is blocked by leptomycin B treatment, most of the Tax-2 molecules are also blocked and co-localize with CIITA in the nucleus, suggesting that CIITA-Tax-2 binding does not preclude Tax-2 entry into the nucleus.

Finally, the nuclear factor NF-YB, also strongly binds to Tax-2. Notably, although endogenous NF-YB does not inhibit Tax-2-dependent HTLV-2 LTR transactivation, it still binds to Tax-2, and in presence of CIITA, this binding seems to increase.

**Conclusions:**

These results strongly suggest that CIITA inhibit Tax-2 by binding the viral transactivator both directly or through a tripartite interaction with NF-YB in. CIITA is therefore a viral restriction factor for HTLV-2 and this open the possibility to control HTLV-2 viral replication and spreading by the controlled induction of CIITA in infected cells

## Background

HTLV-1 (Human T cell Lymphotropic Virus type 1) and HTLV-2 (Human T cell Lymphotropic virus type 2) are closely related human retroviruses that belong to deltaviridae family, subfamily oncovirus type C, characterized by similar genomic organization and common modes of transmission but different disease manifestations [1]. It is estimated that about 15-20 millions of people live with HTLV infection worldwide [2]. HTLV-1 infection is endemic in Japan, Africa, South America, and the Caribbean basin. HTLV-2 infection is highly concentrated in Central and West Africa, in native Amerindian populations in North, Central, and South America, and among cohorts of intravenous drug users (IVDUs) in the United States and Europe [3]. HTLVs are transmitted sexually, by breast feeding or by blood transfusions [4].

HTLV-1 and HTLV-2 show a differential cellular tropism. HTLV-1 has a preferential tropism for CD4^+ ^T cells [5] while HTLV-2 preferentially infects CD8+ T cells, although this restriction is not absolute, as both viruses may also infect B cells, monocytes, microglial and endothelial cells, at least in vitro [6-8]. HTLV-1 is the etiologic agent of adult T-cell leukaemia/lymphoma (ATLL) and of the tropical spastic paraparesis/HTLV-1 associated myelopathy (TSP/HAM) [9-12]. Conversely, no clear association to specific diseases has been described for HTLV-2 infection [1].

The basis of HTLV mediated cellular transformation is not completely understood, but it involves the viral transactivator protein Tax. Tax is essential for HTLV-1- and HTLV-2-mediated immortalization of primary human T cells [13,14] and for tumors induction in transgenic mice [15,16]. The precise mechanism by which Tax initiates the malignant process is unclear, but it seems to involve the de-regulation of several steps both at transcriptional and post-transcriptional level [17]. Tax activates transcription of many cellular genes, including interleukin-2 (IL-2) and IL-2Ra [18,19] and affects critical signal transduction pathways regulating cell cycle, cell growth, DNA repair and apoptosis [20]. Many evidences indicate that the transcriptional activation of cellular genes is mediated by Tax-dependent activation of transcriptional factors, such as CREB/ATF, NF-kB and SRF (Serum Responsive Factor). As Tax plays such an important role in gene expression and pathogenesis of HTLV viruses, numerous studies have been directed toward the understanding of the mechanism of Tax transactivation.

We reported that Tax-2 transactivation of the HTLV-2 LTR is strongly inhibited by the host transcription factor CIITA. As a consequence, susceptible T and B human cells do not support HTLV-2 replication when expressing CIITA [21,22]. Similarly, CIITA targets the viral transactivator Tat to inhibit the replication of the HIV-1 virus [23,24].

The *AIR-1 *locus-encoded class II transactivator CIITA is the master regulator of the expression of Major Histocompatibility Complex class II (MHC-II) genes [25-27]. MHC-II-encoded molecules play a key role in the homeostasis of the immune system. They present peptides to the antigen receptor of CD4+ T cells (TH), whose activation is required to trigger and modulate both humoral and cellular immune responses [28]. CIITA is a non-DNA-binding transcriptional integrator recruited to MHC-II promoters via multiple interactions with transcription factors bound to DNA, including the RFX and the NF-Y complexes [29-34]. It interacts with CBP, p300, PCAF as well as the cyclin T1 subunit of the positive transcription elongation factor b (P-TEFb) to enhance MHC-II gene transcription [35-38]. P-TEFb is also used by Tat to promote the elongation of HIV-1 viral transcripts [39] and we have shown that sequestration of cyclin T1 is the major mechanism by which CIITA blocks the transactivating function of Tat [23]. On the contrary, the molecular basis of the CIITA-mediated inhibition of Tax-2 is still not completely understood. Previous investigations have established that the CIITA 1-321 N-terminal region, with an exclusive nuclear distribution, inhibits Tax-2 function and viral replication. We identified CBP and p300 as crucial factors for the Tax-2-directed LTR transactivation. However, they are not involved in CIITA-mediated inhibition of Tax-2. Instead the overexpression of the ubiquitous transcription factor NF-YB, that interacts with CIITA in the MHC class II enhanceosome, was found to inhibit Tax-2 transactivating function [21].

In this paper we have investigated the intimate molecular nature of the CIITA mediated inhibition of Tax-2. We found that both CIITA and NF-Y interact in vivo with Tax-2. We identified both an N-terminal and a C-terminal region of CIITA interacting with the viral transactivator, although, as stated above, only the N-terminal region is involved in the inhibition of Tax-2 function. Interestingly, in absence of CIITA, endogenous NF-YB can still bind to Tax-2, although, as we have previously shown, this interaction does not results in functional inactivation of Tax-2 on the HTLV-2 LTR promoter. CIITA-NF-YB interaction in vivo is stabilized and/or favoured by the presence of Tax-2. Thus concomitant interaction of Tax-2 with CIITA and NF-YB, most likely in the CIITA-NF-YB molecular complex, is at the basis of the functional inactivation of Tax-2 leading to the inhibition of HTLV-2 retrovirus replication. Further studies of subcellular localization unveiled the co-localization of Tax-2 and CIITA both in the cytoplasm and the nucleus, and the role of CIITA in redirecting, upon binding, Tax-2 molecules mostly in the cytoplasm.

These results are discussed within the present knowledge of cell host-pathogen interaction and the identification of the dual role of CIITA as modulator of adaptive immunity and restriction factor against human retroviruses.

## Methods

### Plasmids

Full length CIITA (pcDNA3flagCIITA1-1130) and deletion mutants of it (pcDNA3flagCIITA1-252, pcDNA3flagCIITA1-321, pcDNA3flagCIITA253-1130, pcDNA3flagCIITA253-410) vectors have been described [40]. The flag tag does not affect protein expression and CIITA capacity to transactivate class II promoters. Tax-2 V5 plasmid was a gift of Prof. Bertazzoni, University of Verona, Italy. NF-YB cDNA (pcDNA3mycNF-YB) has been described [33].

### Transient transfections, Co-Immunoprecipitation and Western blotting

Human embryo-derived kidney cell line 293T was maintained in DMEM supplemented with 10% FCS and 5 mM glutamine at 37°C and 5% CO_2_). 293T cells were transfected with expressing constructs for the full-length Flag-CIITA or Flag-CIITA deletion fragments using Lipofectamine (Invitrogen, by Life technology, UK) following the manufactory protocol. After 24 h, cells were collected, resuspended in lysis buffer (1% NP-40, 10 mM Tris-HCl pH 7.4, 150 mM NaCl, 2 mM EDTA) supplemented with 0,1% protease inhibitor mixture (Aprotin, Bestain, E-64, Leupetin, pepstain A, Sigma Aldrich Italia SRL, Milan, Italy) for 45 min on ice and centrifuged for 15 min, 14.000 rpm at 4°C. After preclearing the extracts with 10 μl of 100% mouse Protein A Sepharose 4 fast flow beads (Amersham Pharmacia, Milan, Italy) for 30 minutes at 4°C by rotation, 0.5 μl of anti-V5 antibody (Invitrogen) were added and the mixture incubated for 1 hour on ice and then reacted with 50 μl of Protein A Sepharose 4 fast flow overnight at 4°C by rotation. Alternatively cell lysates were immunoprecipitated with 50 μl of anti-Flag M2 Affinity Gel (Santa Cruz Biotechnology, Santa Cruz, CA). An aliquot corresponding to 12% of the total cell extract was conserved for proteins expression detection (input). Immunocomplexes were collected by centrifugation, washed five times with the above lysis buffer and once with the lysis buffer containing 500 mM NaCl. The immunocomplexes were detected after SDS-PAGE and Western blotting as described [23] with either the anti-c-Myc antibody (9E10 monoclonal antibody, Santa Cruz Biotechnology, Santa Cruz, CA), the anti-Flag M2 or anti-CIITA 7-1H monoclonal antibodies (Sigma Aldrich), or the anti-NFYB polyclonal rabbit antiserum (Santa Cruz), followed by an HRP-conjugated anti-rabbit or anti-mouse Ig secondary antibody (Amersham Pharmacia, Milan, Italy). To detect Tax-2 V5 protein we used the anti-V5 antibody directly conjugated with HRP (anti-V5-HRP antibody, Sigma Aldrich). Blots were developed by chemiluminescence assay (ECL, Amersham Pharmacia).

### Immunofluorescence staining

Human 293T cells were seeded on glass coverslips and transiently transfected with 1.5 μg of the indicated expression vectors with Lipofectamine (Invitrogen). 24 h post-tranfection the cells were fixed by incubation with 100% methanol at -20 for 6 min. The cells were washed with PBS and blocked for 1 h in PBS containing 0.5% gelatin (Biorad) and 0.5% bovine serum albumine (Sigma), before overnight incubation at 4°C with monoclonal V5 antibodies (Invitrogen) diluited 1:750 in the blocking solution. Goat anti-mouse IgG2a Fab conjugated to Alexa Fluor 546 (Molecular Probes) was used as secondary antibody. Samples were then mounted in Fluor Save reagent (Calbiochem) and analyzed with a laser scanning confocal microscope (Leica) using a 63 × objective and light source wavelengths of 488 and 543 nm.

## Results

### CIITA interacts with Tax-2 in vivo

**Figure 1:**
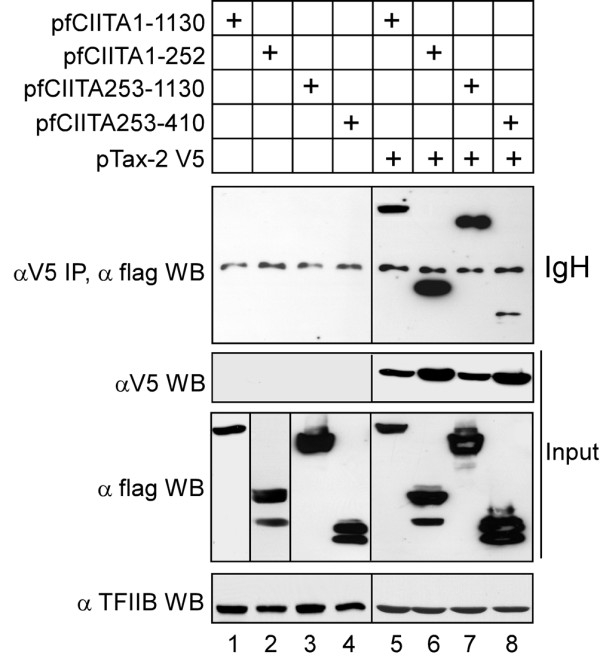
**CIITA interacts with Tax-2 *in vivo***. 293T cells were transiently co-transfected with either one of the following CIITA plasmids pcfCIITA wt (3 μg), pcfCIITA 1-252 (3 μg), pcfCIITA 253-1130 (3 μg), pcfCIITA 253-410 (3 μg), pcfCIITA 1-321(1.5 μg) and pTax-2 V5 (2 μg) vector. Extracts were immunoprecipitated (IP) with the anti-V5 monoclonal antibody and the purified complexes were immunoblotted (WB) with the anti-Flag antibody for the detection of CIITA and its deletion fragments (top panels). The expression of the proteins in all cell extracts was also examined by WB (input) with the anti-Flag antibody. TFIIB was used as a control to show that equal amounts of total protein were loaded in each lane (bottom panels). To be noted, the input expression of the CIITA fragment 1-252 was evaluated in a different western blot gel as underlined by the lines in lane 2. IgH, non specific band representing the immunoglobulin heavy chain of the anti-V5 antibody recognized in western blots after immunoprecipitation and detection with HRP-conjugated anti-rabbit or anti-mouse Ig secondary antibody.

In order to verify whether CIITA-mediated inhibition of Tax-2 could correlate with a direct binding between the two factors, flag-tagged CIITA and V5-tagged Tax-2 were transiently co-expressed in 293T cells. Cell lysates were immunoprecipitated with the anti-V5 antibody and immunocomplexes were examined for the presence of flagCIITA by anti-Flag western blotting. Results clearly indicate that CIITA and Tax-2 strongly interact each other in vivo (Figure 1, top panel, lane 5)

To define the region(s) of CIITA mediating the interaction with the viral transactivator, several truncated forms of CIITA were tested for their ability to bind Tax-2 by co-immunoprecipitation assay in 293T cells. When either CIITA full length or CIITA fragments were expressed in absence of Tax-2 no specific bands were detected. A non specific band of 55 kD was present in all immunoprecipitates (Figure 1A, top panel, lanes 1-8).

The N-term fragment fCIITA 1-252 interacted strongly with Tax-2 (Figure 1, top panel, lane 6). Interestingly, also the complementary C-term fragment 253-1130 interacted with Tax-2 (Figure 1A, top panel, lane 7). An overlapping N-term fragment fCIITA 253-410, although well expressed after transfection, (Figure 1A, lower panel input, lane 8) only slightly interacted with Tax-2 (Figure 1A, top panel, lane 8) as compared to the fCIITA 1-252 and fCIITA 253-1130 fragments. These results indicate a complex pattern of interaction between CIITA and Tax-2 with at least two regions of CIITA, encompassing the N-term1-252 and at the C-term 410-1130 of the molecule, respectively, strongly interacting with the viral transactivator, although we cannot exclude that residues included in the 253-410 region, themselves very weakly interacting with Tax2, may participate in generating the correct conformation for the critical binding site of the strongly interacting 253-1130 CIITA fragment.

### The CIITA N-term 1-252, but not the C-term 253-1130, region inhibits Tax-2 transactivating activity in 293T cells

It has been previously shown that the N-terminal region of CIITA mediates the inhibition of Tax-2-

dependent HTLV-2 LTR transactrivation in COS-7 cells [21,22]. As the interaction studies described here were performed instead in human 293T cells, and at least two regions were shown to interact with Tax-2, it was important to assess the pattern of CIITA-mediated inhibition of Tax-2 function in these cells, representative of the species naturally infected by HTLV-2

To this end cells were co-transfected with a fixed amount of the Tax-2 expression vector (pTax-2 V5) and increasing amounts of plasmids encoding CIITA wild type (pfCIITA 1-1130), N-term (pfCIITA 1-252, or pfCIITA 1-321) or C-term (pf253-1130) fragments, respectively. It must be noted that in this assay 10 fold less DNA was transfected into the cells as compared to the interaction mapping of Figure 1.

**Figure 2:**
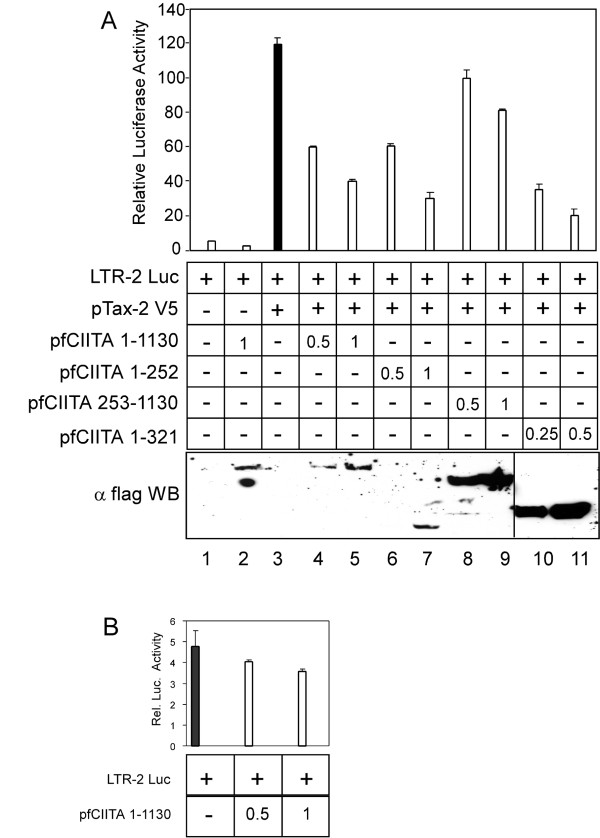
**The CIITA N-term 1-252, but not the C-term 253-1130, region inhibits Tax-2 transactivating activity in 293T cells**. A)- Luciferase gene reporter assay performed in 293T cells transiently co-transfected with fixed amounts (0.2 μg) of pLTR-II-Luc and pcTax-2 V5.(0.05 μg) vectors and in the absence or presence of increasing amounts (0.5-1 μg) of vectors coding for Flag-tagged CIITA wt (pfCIITA 1-1130) and fragments (pfCIITA 1-252 and pfCIITA 253-1130). Lower amounts of vector coding for flagCIITA 1-321 fragment (0.25-0.5 μg) were transfected. The black histogram represents the LTR-2 promoter activation by Tax-2 (bar 3). Bar 1 represents the control activity of the pcDNA3 vector alone. B)- CIITA did not affect basal promoter activity even after transfection of increasing amounts of CIITA plasmid. The expression of recombinant fCIITA proteins in all cell extracts were detected by anti-Flag Western blot (WB) (bottom panel).

Results show that Tax-2-mediated activation of the viral LTR promoter (Figure 2A, bar 3) was significantly inhibited by CIITA wild type (Figure 2A, bars 4 and 5) and the N-term 1-252 and 1-321 CIITA fragments (Figure 2A, bars 6-7 and 10-11, respectively) in a dose-dependent manner, whereas the C-term 253-1130 fragment (bars 8-9) exerted only a modest inhibition on Tax-2 activity. CIITA wt expression vector did not significantly affect basal promoter activity (Figure 2A, bar 2; Figure 2B).

Thus, also in 293T cells CIITA-dependent inhibition of Tax-2 function correlates with the N-term 1-252 region of CIITA. Furthermore, these results strongly suggest that interaction between the N-terminal, but not the C-terminal, part of CIITA, and Tax-2 is responsible of the biological effect of CIITA on the viral transactivator.

### Tax-2 and NF-YB interact in vivo

Previous results from our laboratory have shown that the ubiquitously expressed nuclear transcription factor NF-YB, which interacts and co-operates with CIITA in activating HLA-II genes transcription, could inhibit the HTLV-2 LTR promoter transactivation by Tax-2 in COS-7 cells when over-expressed after transfection [22]. Similar experiments performed in 293T cells resulted in comparable findings (data not shown).

**Figure 3:**
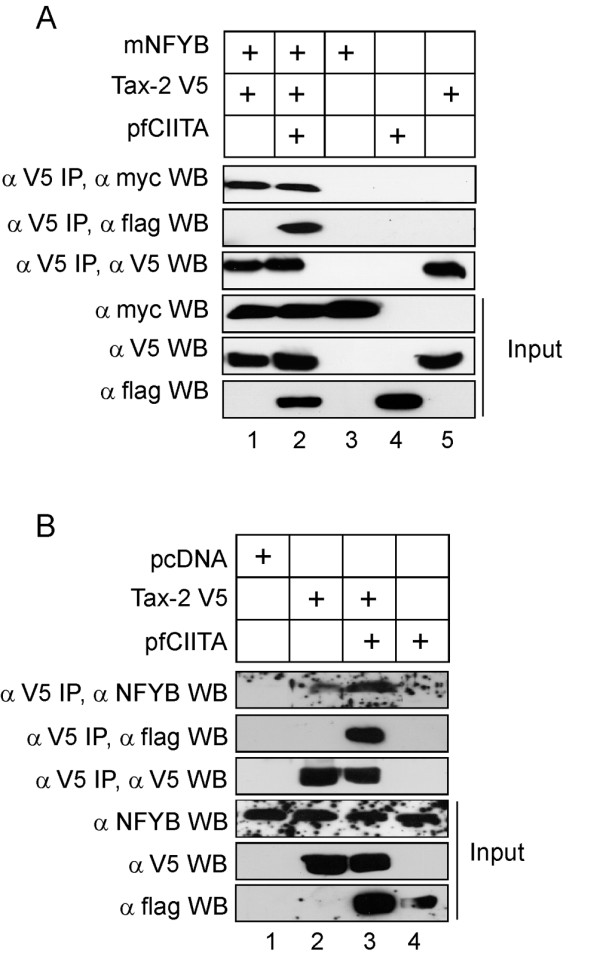
**Tax-2 and NF-YB interact *in vivo***. Panel A- 293T cells were transiently co-transfected with pcMycNF-YB (2 ug), pcTax-2 V5 (4 ug) and pcfCIITA (3 ug) vectors. Cell extracts were immunoprecipitated (IP) with the anti-V5 monoclonal antibody and the purified complexes were immunoblotted (WB) with the indicated antibodies for the detection of NFYB and CIITA. The expression of the proteins in whole cell extracts was also examined by WB (input) with antibodies directed against myc, Flag and V5. Panel B. 293T cells were transiently transfected with pcTax-2 V5 (4 ug), pcfCIITA (3 ug) and/or the empty vector pcDNA and immunoprecipitated as in A. The purified immunocomplexes were immunoblotted with the anti-NFYB and the anti-Flag antibodies for the detection of the endogenous NFYB and of CIITA, respectively. The expression of the proteins in whole cell extracts was also examined by WB (input) with antibodies directed against NFYB, Flag and V5.

In order to investigate whether NF-YB could also interact with Tax-2 *in vivo *we performed initially co-immunoprecipation experiments by using lysates of 293T cells co-transfected with myc-tagged NF-YB (mNFYB) and V5-tagged Tax-2. Results presented in Figure 3A show that Tax-2 interacts with NF-YB not only in the presence of co-transfected CIITA (Figure 3A, αV5 IP, αflag WB, lane 2), but also in the absence of CIITA (Figure 3A, αV5 IP, αmyc WB, lane 1). Experiments were then carried out to assess whether endogenous NF-YB could interact *in vivo *with Tax-2. Although with the limitations of the relatively low expression of the endogenous NF-YB protein with respect to the protein expressed after transient transfection, the interaction of NF-YB with Tax-2 was observed also in this case (Figure 3B, αV5 IP, αNFYB WB, lane 2). Interestingly, in the presence of co-transfected CIITA, the amount of co-immunoprecipitated endogenous NF-YB with Tax-2 was clearly increased (Figure 3B, αV5 IP, αflag WB, lane 3). Taken together, these results indicate that NF-YB interacts with Tax-2 and this interaction can be increased and/or stabilized by the concomitant interaction with CIITA, leading to functional impairment of Tax-2 function.

### Subcellular distribution of Tax-2 in presence of CIITA

In order to obtain a deeper insight into the mechanism of CIITA-mediated inhibition on Tax-2 function, the subcellular distribution of Tax-2 molecules was analyzed in the presence and in the absence of CIITA.

**Figure 4:**
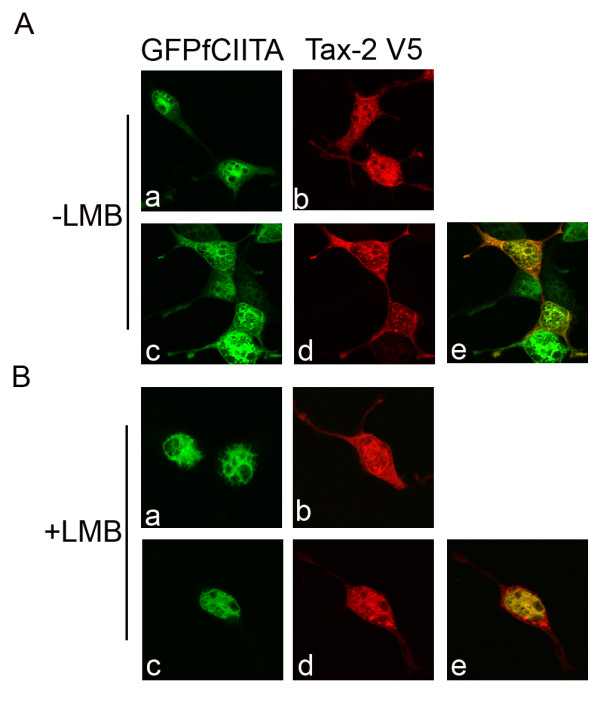
**CIITA affects Tax-2 subcellular localization**. 293T cells were transiently transfected with the indicated vectors (GFPCIITA, Tax2-V5). Eighteen hours post transfection, cells were treated (B) or not (A) with leptomycin B (20 nm) for 3 hours. Cells were then washed, fixed, and stained with anti-V5 IgG2a monoclonal antibody for the detection of V5-tagged Tax proteins (Ab, Ad, Bb, Bd). GFPCIITA positive cells are shown in Aa, Ac, Ba, Bc. Merged images are shown in Ae, Be. The images were analyzed by a laser scanning confocal microscope.

In the absence of CIITA, Tax-2 localizes both and in the cytoplasm and in the nucleus of 293T cells often with a punctuated aspect (Figure 4A, panel b). Similarly, CIITA in the absence of Tax-2, localized in both compartments, with a predominant nuclear distribution and in more diffused aspect as compared to the punctuated Tax-2 distribution (Figure 4A, panel a).

In the presence of CIITA, Tax-2 is predominantly accumulated in the cytoplasm and with a marked staining around the nuclear membrane where it formed a ring-like structure (Figure 4A, panel d). Interestingly, an overlapping co-localization of Tax-2 and CIITA was observed in the cytoplasm as well as in perinuclear ring (Figure 4A, panel e).

CIITA contains both nuclear import (NIS) and nuclear export (NES) signals that allow the molecule to shuttle between cytoplasm and nucleus. CIITA NES are CRM-1 dependent, as treatment of CIITA-positive cells with the CRM-1 inhibitor leptomycin B (LMB) relocalizes CIITA mostly within the nucleus [41,42]. HTLV-2 Tax-2 protein also contain NIS and most likely NES although the latter have been demonstrated not to be CRM-1-dependent in HeLa cells [43]. It was therefore important to assess whether Tax-2 subcellular distribution in 293T cells could be modified by LMB treatment in presence or in absence of CIITA. The results presented in Figure 4B show that LMB treated CIITA-transfected cells displayed, as expected, an exclusive CIITA nuclear localization (Figure 4B, panel a). On the other hand, LMB-treated Tax-2 transfected cells displayed a cytoplasmatic and nuclear distribution very similar to that of untreated cells (Figure 4B, panel b). In CIITA and Tax-2 co-transfected cells treated with LMB, again CIITA was exclusively in the nucleus (Figure 4B, panel c). Interestingly, in this case Tax-2 was also predominantly localized in the nucleus (Figure 4B panel d) and a strong nuclear co-localization of the two proteins was observed (Figure 4B panel e).

Taken together, the above subcellular localization studies indicate that the physical CIITA-Tax-2 interaction is mirrored by a strong co-localization of the two molecules in cytoplasmic and nuclear subcellular compartments. Moreover and of particular importance, interaction with CIITA makes Tax-2 molecules prone to migrate to cytoplasm where they can no longer exert their transactivating function on the HTLV-2 LTR.

## Discussion

Host-pathogen interaction is regulated by a series of cellular and molecular mechanisms whose outcome dictates in many instances the subtle equilibrium between control of infection and pathological consequences for the host. This is particular relevant for pathogens like human oncogenic retroviruses such as HTLVs whose infectivity can generate, as clearly demonstrated for HTLV-1, not only severe infections but also neoplastic transformation [17]. It is therefore important to investigate possible molecular interactions between host-derived and virus-derived factors as a necessary framework to understand the evolution of infection. Previous investigation from our laboratory has demonstrated that the MHC class II transactivator CIITA could block the replication of the human HTLV-2 retrovirus by inhibiting the function of the viral transactivator protein Tax-2 [21,22]. However the biochemical basis of the CIITA-mediated inhibition on Tax-2 function was not clarified. In the present investigation we focused our analysis on this specific aspect, trying to understand whether CIITA-mediated inhibition requires a physical interaction between the cellular protein and the viral transactivator. Moreover, as the transcription factor NF-YB, whose interaction with CIITA is necessary for MHC class II gene transcription, was also shown to inhibit Tax-2 when overexpressed in COS-7 cells, we investigated whether NF-YB and Tax-2 physically interacted *in vivo*.

We demonstrated for the first time the existence of *in vivo *interaction between CIITA and Tax-2. (Figure 1) and shown that this interaction is quite complex as it involves at least two region of the CIITA molecule located at the 1-252 N-terminal and mostly at the 410-1130 C-terminal site. Interestingly, however, and in similarity to the results obtained in COS-7 cells [22], only the N-terminal CIITA region was able to functionally inhibit Tax-2 dependent HTLV-2 LTR transactivation in 293T cells. It is of note that the N-terminal region of CIITA is the one that mostly interacts with nuclear factors that bind the MHC class II gene promoter region, including general transcription factors regulating initiation of transcription, chromatin modulating factors and NF-YB subunit of the NF-Y trimeric complex [34]. It is therefore of particular relevance the finding presented here that also the previously reported inhibition of Tax-2-mediated LTR transactivation by overexpression of NF-YB, correlated with a strong binding *in vivo *between Tax-2 and NF-YB. In fact, and again for the first time, we demonstrated in this investigation that both transfected, thus overexpressed, and endogenous NF-YB could interact with the viral transactivator. Whether this is a direct interaction or requires a third partner is presently under scrutiny. Moreover the binding of endogenous NF-YB with Tax-2 molecules was increased by the presence of CIITA, strongly suggesting that at least a trimeric NF-YB-CIITA-Tax-2 complex is formed *in vivo*, in the nucleus and this could be a mechanism preventing Tax-2 from correctly transactivate HTLV-2 LTR and thus viral replication. It remains to be established whether the binding of the viral transactivator with CIITA and NF-YB, prevents Tax-2 from interacting with its own promoter or the Tax-2 -LTR promoter complex is formed but is functionally inhibited by a mechanism of steric hindrance.

Although the Tax-2-dependent HTLV-2 LTR transactivation takes place in the nucleus, it is still possible that additional mechanisms of CIITA-mediated inhibition of Tax-2 function operate outside the nucleus. For this reason, experiments of subcellular Tax-2 localization in the presence of CIITA were performed by immunofluorescence and confocal microscopy. In the absence of CIITA, Tax-2 localized both in the cytoplasm and the nucleus in the great majority of the cells. These results obtained in 293T cells are only partially similar to those obtained by other groups, using other cellular systems, as for example both in HeLa and in HEP-2 cells Tax-2 was mostly localized in the cytoplasm [44,45]. This discrepancy could be due to the biological properties of the different cell lines analyzed [46]. In the presence of CIITA, the amount of cytoplasmic Tax-2 was visibly increased and the viral transactivator strongly co-localized with CIITA diffusely in the cytoplasm and around the nuclear membrane in a ring-like fashion. While the ring-like distribution has been previously observed for Tax2 colocalizing with calreticulin in a different cell system [45], no description of such a localization for CIITA has been previously reported. Thus, it is likely that Tax2-CIITA interaction generates either by itself or, most likely, via the interaction with other proteins the perinuclear ring-like distribution observed, whose biochemical and functional meaning remains to be elucidated. Interestingly, although reduced in concentration, also the nuclear Tax-2 co-localized with CIITA. Thus, interaction with CIITA makes Tax-2 more prone to segregate into the cytoplasm. Is this a mechanism to prevent Tax-2 from migrating into the nucleus and thus exerting its transactivating function on the HTLV-2 promoter? Two experimental evidences were against this possibility. First, Tax-2 can bind NF-YB which displays a predominant, if not exclusive, nuclear distribution [47]. Second, treatment of the cells with leptomycin B (LMB) which prevents CRM-1-dependent nuclear export resulted in a very prominent CIITA and Tax-2 retention and co-localization in the nucleus. These results strongly suggest that CIITA-Tax-2 complexes, wherever they form, shuttle between nucleus and cytoplasm where they preferentially accumulate, and it is the formation of the complex, and not the subcellular localization that makes Tax-2 functionally incompetent in activating the HTLV-2 LTR promoter. Within this frame the relative contribution of Tax-2-endogenous NF-YB in vivo interaction to the functional impairment of Tax-2 is difficult to assess, because NF-YB is an ubiquitous factor and only part of the nuclear Tax-2 molecules are bound by this factor, as demonstrated by the fact that in presence of CIITA a proportion of nuclear Tax-2 molecules can shuttle to the cytoplasm. At the present NF-YB knock-out cells are not available to study specifically the above aspect. Future experiments, possibly by using siRNA technology, will help to clarify this issue.

## Conclusions

The direct interaction of CIITA with Tax-2, a crucial regulator of human oncogenic retrovirus replication, opens new ways for understanding the peculiar mechanisms by which CIITA has evolved its dual function to counteract pathogens' infections. From one side, CIITA triggers the molecular events leading to transcription of MHC class II genes, whose encoded molecules serve as antigen presenting receptors for peptides from all sort of pathogens, including viruses. In so doing CIITA governs the CD4+ T cell triggering leading to optimal activation of immune effector mechanisms, particularly specific antibody production by B cells. Antibody binding is a crucial event for neutralization of extracellular viruses which cannot infect host cells and are driven to degradation. From the other side, the newly acquired function of CIITA as a molecule that directly binds HTLV-2 Tax-2, and physically neutralizes its activating function on viral replication, represents a potent mechanism of intrinsic immunity. Together with previous results of our group demonstrating the inhibition of HIV-1 retrovirus replication by CIITA [23], the results presented in this study definitively identifies CIITA as an important viral restriction factor for human retroviruses. The results of this study may contribute to envisage novel therapeutic strategies aimed at counteracting retroviral infections through the control of CIITA expression and/or the selective use of CIITA fragments.

## Competing interests

The authors declare that they have no competing interests.

## Authors' contributions

CO carried out the biochemical studies, immunoassays, participated in the discussion of results and drafted the manuscript. GF carried out immunofluorescence staining assay, participated in the discussion of results and drafted the manuscript. GT participated in the design and coordination of the study and in the discussion of the results. RSA conceived the study, and participated in its design and coordination and drafted the manuscript. All authors read and approved the final manuscript.
